# Poly[(μ_3_-5-amino­isophthalato-κ^4^ *O*,*O*′:*O*′′:*O*′′′)[μ_2_-1,2-bis­(4-pyrid­yl)ethane-κ^2^ *N*:*N*′]cobalt(II)]

**DOI:** 10.1107/S1600536810030710

**Published:** 2010-08-11

**Authors:** Shie Fu Lush, Fwu Ming Shen

**Affiliations:** a General Education Center, Yuanpei University, Hsinchu 30015, Taiwan; b Department of Biotechnology, Yuanpei University, No. 306, Yuanpei St., Hsinchu 30015, Taiwan

## Abstract

In the title compound, [Co(C_8_H_5_NO_4_)(C_12_H_12_N_2_)]_*n*_, the Co^II^ ion presents a distorted CoO_4_N_2_ octa­hedral coordination geometry, formed by three 5-amino­isophthalate dianions and two 1,2-bis­(4-pyrid­yl)ethane ligands. One carboxyl­ate group of the 5-amino­isophthalate dianion chelates a Co cation and the other carboxyl­ate group bridges the other two Co cations, while the terminal N atoms of the 1,2-bis­(4-pyrid­yl)ethane ligand coordinate the neighboring Co cations, forming a two-dimensional polymeric architecture. Two pyridine rings of the 1,2-bis­(4-pyrid­yl)ethane ligand are twisted to each other with a dihedral angle of 50.94 (16)°. Weak C—H⋯O hydrogen bonding and N—H⋯π inter­actions are observed in the crystal structure. A void of 69 (5) Å^3^ is present in the crystal structure, but no solvent mol­ecule can be located reasonably.

## Related literature

For similar polymeric structures, see: He *et al.* (2006 ▶); Tang *et al.* (2007 ▶); Zhang *et al.* (2007 ▶); Ou *et al.* (2008 ▶); Zhang *et al.* (2009 ▶).
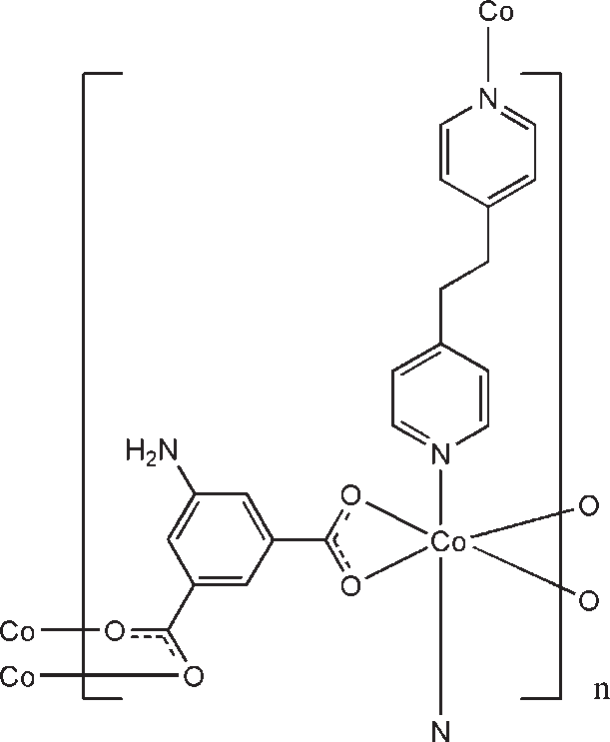


## Experimental

### 

#### Crystal data

[Co(C_8_H_5_NO_4_)(C_12_H_12_N_2_)]
*M* *_r_* = 422.30
Triclinic, 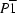

*a* = 9.9093 (2) Å
*b* = 10.0755 (2) Å
*c* = 10.5065 (3) Å
α = 78.301 (1)°
β = 83.560 (1)°
γ = 68.074 (2)°
*V* = 952.12 (4) Å^3^
*Z* = 2
Mo *K*α radiation
μ = 0.93 mm^−1^
*T* = 298 K
0.22 × 0.18 × 0.08 mm

#### Data collection

Nonius KappaCCD diffractometer
Absorption correction: multi-scan (*SCALEPACK*; Otwinowski & Minor, 1997 ▶) *T*_min_ = 0.732, *T*_max_ = 0.840
7715 measured reflections
3356 independent reflections
3003 reflections with *I* > 2σ(*I*)
*R*_int_ = 0.038

#### Refinement

*R*[*F* ^2^ > 2σ(*F* ^2^)] = 0.035
*wR*(*F* ^2^) = 0.120
*S* = 1.16
3356 reflections
253 parameters
H-atom parameters constrained
Δρ_max_ = 0.58 e Å^−3^
Δρ_min_ = −0.60 e Å^−3^

### 

Data collection: *COLLECT* (Nonius, 2000 ▶); cell refinement: *SCALEPACK* (Otwinowski & Minor, 1997 ▶); data reduction: *DENZO* (Otwinowski & Minor, 1997 ▶) and *SCALEPACK*; program(s) used to solve structure: *SHELXS97* (Sheldrick, 2008 ▶); program(s) used to refine structure: *SHELXL97* (Sheldrick, 2008 ▶); molecular graphics: *PLATON* (Spek, 2009 ▶); software used to prepare material for publication: *PLATON*.

## Supplementary Material

Crystal structure: contains datablocks global, I. DOI: 10.1107/S1600536810030710/xu5004sup1.cif

Structure factors: contains datablocks I. DOI: 10.1107/S1600536810030710/xu5004Isup2.hkl

Additional supplementary materials:  crystallographic information; 3D view; checkCIF report

## Figures and Tables

**Table 1:** Selected bond lengths (Å)

Co1—N1	2.178 (2)
Co1—N2^i^	2.175 (3)
Co1—O1	2.0416 (18)
Co1—O2^ii^	2.011 (2)
Co1—O3^iii^	2.1426 (19)
Co1—O4^iii^	2.228 (2)

Symmetry codes: (i) 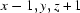; (ii) 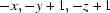; (iii) 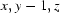.

**Table 2:** Hydrogen-bond geometry (Å, °) *Cg*4 is the centroid of the N1-pyridine ring.

*D*—H⋯*A*	*D*—H	H⋯*A*	*D*⋯*A*	*D*—H⋯*A*
C4—H4⋯O4^iv^	0.93	2.35	3.271 (4)	173
C10—H10⋯O2^v^	0.93	2.44	3.276 (5)	150
C15—H15⋯O3^vi^	0.93	2.56	3.487 (4)	175
N3—H3*A*⋯*Cg*4^vii^	0.86	2.92	3.765 (3)	169

Symmetry codes: (iv) 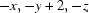; (v) 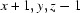; (vi) 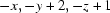; (vii) 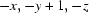.

**Fig. 1.**
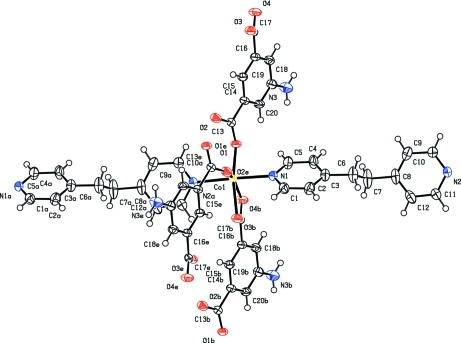
View of the title compound with the atom numbering scheme. Displacement ellipsoids for non-H atoms are drawn at the 50% probability level.

## supplementary crystallographic information

### Comment

In recent years, we have been focused on organic-inorganic hybrid material containing either N– or O-donor rigid heteroaromatic ligands, such as 5-Aminoisophthalic acid (aip). The polycarboxylic acid ligands can bridge one or more metal centers and produce neutral architectures. Hence, metal-organic coordination polymers constructed by mixed ligands of pyridyl and carboxylate groups not only incorporate interesting propertied of different functional groups but also are more adjustable through changing one of the mixing organic ligands. However, few coordination polymers based on amino aromatic di or poly(carboxylic acids) ligands and bipyridine has been reported (He *et al.* 2006; Tang *et al.* 2007; Zhang *et al.* 2007; Ou *et al.* 2008; Zhang *et al.* 2009).

The title compound by X-ray crystallography reveals that the symmetric unit consist of one Co^II^ ion, two 1,2-bis(4-pyridyl)ethane (dpe) ligands and three aip ligands, as shown in Fig. 1. The Co^II^ ion is six-coordinated with a slightly distorted octahedral geometry. The equatorial plane is occupied by two monodentate carboxylate oxygen atoms from two aip ligands and one bidentate carboxylate oxygen atoms from one aip ligand, while the axial sites are occupied by two nitrogen atoms of the pyridine groups from two dpe ligands (Table 1). Each aip ligand employs its two carboxylate groups in turn to coordinate to three metal centers, while the remains amino group in uncoordinated manner. The four symmetry-related metal centers are linked by two aip ligands and two dpe ligands to form a 30-membered macro cycle with Co···Co separation of 6.956 (3) Å and 13.610 (8) Å, respectively, showing 1-D open channels along the crystallographic *c* axis. In title polymer, there are no classical hydrogen bonding interactions, but C—H···O hydrogen-bonding is observed in the crystal structure (Table 2).

In addition, C—H···π interactions C11—H11···*Cg*3 (N1/C2—C5), N—H···π interactions N3—H···*Cg*4 (N2/C8—C12) are present in the crystal structure (full details and symmetry codes are given in Table 2). π···π stacking interactions are also observed, the centroid-centroid between *Cg*3(O3—O4/C17/Co1c)···*Cg*4^vii^(N1/C1—C5), *Cg*3···*Cg*5(N2/C8—C12)^viii^ are 3.8307 (17) and 3.9143 (18) [symmetry codes: (vii)= *X*, 1+Y, *Z*, (viii)= 1-*X*,1-Y,-*Z*.], respectively.

### Experimental

CoBr_2_ (0.1097 g, 0.5 mmol), 5-aminoisophthalic acid, (0.0903 g, 0.5 mmol) and 1,2-bis(4-pyridyl)ethane (0.0913 g, 0.5 mmol) were mixed in 10 ml deionized water. After being stirred for 30 min, the mixture was placed in a 25 ml Teflon liner reactor and heated at 423 K in the oven for 24 h. The resulting solution was slowly cooled to room temperature. The purple transparent single crystals of the title compound were obtained in 46.45% yield (based on cobalt).

### Refinement

H atoms were positioned geometrically with N—H = 0.86, C—H = 0.93 (aromatic) and 0.97 Å (methylene), and were refined using a riding model with U_iso_(H) = 1.2U_eq_(C,N). A voids of 69 Å^3^ exists close to an inversion center in the crystal structure, a solvent water molecule with a fractional site occupancy factor was tried to located, however the refinement including the water molecule gave an abnormal large displacement paramenter and small SOF.

### Crystal data

**Table:**

[Co(C_8_H_5_NO_4_)(C_12_H_12_N_2_)]	*Z* = 2
*M**_r_* = 422.30	*F*(000) = 434
Triclinic, *P*1	*D*_x_ = 1.473 Mg m^−^^3^
Hall symbol: -P 1	Mo *K*α radiation, λ = 0.71073 Å
*a* = 9.9093 (2) Å	Cell parameters from 6854 reflections
*b* = 10.0755 (2) Å	θ = 2.0–25.0°
*c* = 10.5065 (3) Å	µ = 0.93 mm^−^^1^
α = 78.301 (1)°	*T* = 298 K
β = 83.560 (1)°	Prism, purple
γ = 68.074 (2)°	0.22 × 0.18 × 0.08 mm
*V* = 952.12 (4) Å^3^	

### Data collection

**Table:**

Nonius KappaCCD diffractometer	3356 independent reflections
Radiation source: fine-focus sealed tube	3003 reflections with *I* > 2σ(*I*)
graphite	*R*_int_ = 0.038
Detector resolution: 9 pixels mm^-1^	θ_max_ = 25.0°, θ_min_ = 2.2°
ω/2θ scans	*h* = −11→11
Absorption correction: multi-scan (*SCALEPACK*; Otwinowski & Minor, 1997)	*k* = −11→11
*T*_min_ = 0.732, *T*_max_ = 0.840	*l* = −12→12
7715 measured reflections	

### Refinement

**Table:**

Refinement on *F*^2^	Primary atom site location: structure-invariant direct methods
Least-squares matrix: full	Secondary atom site location: difference Fourier map
*R*[*F*^2^ > 2σ(*F*^2^)] = 0.035	Hydrogen site location: inferred from neighbouring sites
*wR*(*F*^2^) = 0.120	H-atom parameters constrained
*S* = 1.16	*w* = 1/[σ^2^(*F*_o_^2^) + (0.0694*P*)^2^ + 0.2452*P*] where *P* = (*F*_o_^2^ + 2*F*_c_^2^)/3
3356 reflections	(Δ/σ)_max_ < 0.001
253 parameters	Δρ_max_ = 0.58 e Å^−^^3^
0 restraints	Δρ_min_ = −0.60 e Å^−^^3^

### Special details

**Table:**

Geometry. Bond distances, angles *etc*. have been calculated using the rounded fractional coordinates. All su's are estimated from the variances of the (full) variance-covariance matrix. The cell e.s.d.'s are taken into account in the estimation of distances, angles and torsion angles
Refinement. Refinement on *F*^2^ for ALL reflections except those flagged by the user for potential systematic errors. Weighted *R*-factors *wR* and all goodnesses of fit *S* are based on *F*^2^, conventional *R*-factors *R* are based on *F*, with *F* set to zero for negative *F*^2^. The observed criterion of *F*^2^ > σ(*F*^2^) is used only for calculating -*R*-factor-obs *etc*. and is not relevant to the choice of reflections for refinement. *R*-factors based on *F*^2^ are statistically about twice as large as those based on *F*, and *R*-factors based on ALL data will be even larger.

### Fractional atomic coordinates and isotropic or equivalent isotropic displacement parameters (Å^2^)

**Table:**

	*x*	*y*	*z*	*U*_iso_*/*U*_eq_	
Co1	−0.03642 (4)	0.35863 (3)	0.37674 (3)	0.0234 (1)	
O1	−0.1184 (2)	0.57793 (18)	0.31360 (18)	0.0285 (6)	
O2	−0.0962 (2)	0.6739 (2)	0.47933 (18)	0.0323 (6)	
O3	0.0094 (2)	1.1365 (2)	0.36324 (18)	0.0308 (6)	
O4	−0.1437 (2)	1.3060 (2)	0.2285 (2)	0.0359 (6)	
N1	0.1467 (2)	0.3521 (2)	0.2393 (2)	0.0279 (7)	
N2	0.7840 (3)	0.3556 (3)	−0.4846 (2)	0.0317 (7)	
N3	−0.2170 (3)	0.9890 (3)	−0.0716 (2)	0.0406 (9)	
C1	0.2784 (3)	0.2469 (3)	0.2530 (3)	0.0358 (9)	
C2	0.3902 (3)	0.2352 (3)	0.1606 (3)	0.0395 (9)	
C3	0.3706 (3)	0.3366 (3)	0.0468 (3)	0.0355 (9)	
C4	0.2349 (3)	0.4469 (4)	0.0337 (3)	0.0423 (10)	
C5	0.1282 (3)	0.4506 (3)	0.1293 (3)	0.0381 (9)	
C6	0.4901 (3)	0.3287 (4)	−0.0575 (3)	0.0493 (10)	
C7	0.4432 (4)	0.3491 (5)	−0.1921 (3)	0.0622 (16)	
C8	0.5608 (3)	0.3512 (4)	−0.2951 (3)	0.0434 (10)	
C9	0.5940 (4)	0.4743 (4)	−0.3398 (4)	0.0577 (12)	
C10	0.7050 (4)	0.4714 (4)	−0.4323 (3)	0.0490 (11)	
C11	0.7517 (3)	0.2363 (3)	−0.4412 (3)	0.0435 (10)	
C12	0.6425 (4)	0.2314 (4)	−0.3494 (3)	0.0491 (11)	
C13	−0.1119 (3)	0.6807 (3)	0.3616 (3)	0.0247 (8)	
C14	−0.1282 (3)	0.8214 (3)	0.2696 (3)	0.0252 (8)	
C15	−0.1013 (3)	0.9306 (3)	0.3130 (3)	0.0269 (8)	
C16	−0.1136 (3)	1.0592 (3)	0.2279 (3)	0.0266 (8)	
C17	−0.0809 (3)	1.1747 (3)	0.2751 (3)	0.0267 (8)	
C18	−0.1542 (3)	1.0802 (3)	0.1017 (3)	0.0295 (8)	
C19	−0.1780 (3)	0.9694 (3)	0.0556 (3)	0.0289 (8)	
C20	−0.1643 (3)	0.8411 (3)	0.1413 (3)	0.0274 (8)	
H1	0.29510	0.17810	0.32900	0.0430*	
H2	0.47920	0.15920	0.17450	0.0470*	
H3A	−0.23300	0.92110	−0.09820	0.0490*	
H3B	−0.22510	1.06900	−0.12380	0.0490*	
H4	0.21640	0.51860	−0.04030	0.0510*	
H5	0.03820	0.52540	0.11730	0.0460*	
H6A	0.52950	0.40260	−0.05370	0.0590*	
H6B	0.56780	0.23490	−0.03870	0.0590*	
H7A	0.36110	0.43990	−0.20910	0.0750*	
H7B	0.41040	0.27120	−0.19800	0.0750*	
H9	0.54140	0.55930	−0.30760	0.0690*	
H10	0.72570	0.55580	−0.45960	0.0590*	
H11	0.80580	0.15250	−0.47470	0.0520*	
H12	0.62370	0.14580	−0.32390	0.0590*	
H15	−0.07540	0.91760	0.39830	0.0320*	
H18	−0.16590	1.16830	0.04670	0.0350*	
H20	−0.17950	0.76670	0.11240	0.0330*	

### Atomic displacement parameters (Å^2^)

**Table:**

	*U*^11^	*U*^22^	*U*^33^	*U*^12^	*U*^13^	*U*^23^
Co1	0.0327 (2)	0.0192 (2)	0.0210 (2)	−0.0133 (2)	0.0040 (2)	−0.0048 (2)
O1	0.0409 (10)	0.0202 (9)	0.0278 (10)	−0.0143 (8)	−0.0001 (8)	−0.0062 (8)
O2	0.0487 (11)	0.0302 (11)	0.0229 (10)	−0.0211 (9)	−0.0035 (9)	−0.0009 (8)
O3	0.0425 (11)	0.0253 (10)	0.0294 (10)	−0.0169 (8)	−0.0038 (9)	−0.0052 (8)
O4	0.0529 (12)	0.0208 (10)	0.0365 (11)	−0.0157 (9)	−0.0079 (10)	−0.0025 (8)
N1	0.0343 (12)	0.0288 (12)	0.0216 (11)	−0.0134 (10)	0.0039 (9)	−0.0057 (9)
N2	0.0359 (12)	0.0333 (13)	0.0279 (12)	−0.0167 (10)	0.0082 (10)	−0.0067 (10)
N3	0.0644 (17)	0.0355 (14)	0.0287 (13)	−0.0250 (13)	−0.0137 (12)	−0.0003 (11)
C1	0.0415 (16)	0.0321 (16)	0.0280 (15)	−0.0108 (13)	0.0031 (13)	0.0001 (12)
C2	0.0342 (15)	0.0410 (17)	0.0355 (17)	−0.0062 (13)	0.0022 (13)	−0.0059 (14)
C3	0.0336 (15)	0.0454 (18)	0.0281 (15)	−0.0158 (13)	0.0058 (12)	−0.0088 (13)
C4	0.0431 (17)	0.0459 (18)	0.0262 (15)	−0.0104 (14)	0.0074 (13)	0.0040 (13)
C5	0.0336 (15)	0.0386 (17)	0.0305 (16)	−0.0043 (12)	0.0056 (12)	−0.0011 (13)
C6	0.0356 (16)	0.072 (2)	0.0366 (18)	−0.0191 (16)	0.0097 (14)	−0.0081 (16)
C7	0.0402 (18)	0.116 (4)	0.0359 (19)	−0.037 (2)	0.0131 (15)	−0.016 (2)
C8	0.0347 (15)	0.071 (2)	0.0256 (15)	−0.0241 (16)	0.0068 (13)	−0.0057 (15)
C9	0.063 (2)	0.062 (2)	0.054 (2)	−0.0273 (19)	0.0290 (18)	−0.0309 (19)
C10	0.060 (2)	0.0447 (19)	0.053 (2)	−0.0313 (16)	0.0259 (17)	−0.0232 (16)
C11	0.0495 (18)	0.0369 (17)	0.0438 (19)	−0.0200 (14)	0.0148 (15)	−0.0068 (14)
C12	0.0530 (19)	0.049 (2)	0.046 (2)	−0.0283 (16)	0.0121 (16)	0.0020 (16)
C13	0.0282 (12)	0.0216 (13)	0.0252 (14)	−0.0116 (10)	0.0023 (10)	−0.0031 (11)
C14	0.0314 (13)	0.0192 (13)	0.0269 (14)	−0.0122 (10)	0.0006 (11)	−0.0029 (10)
C15	0.0379 (14)	0.0213 (13)	0.0231 (13)	−0.0121 (11)	−0.0015 (11)	−0.0044 (10)
C16	0.0344 (13)	0.0210 (13)	0.0269 (14)	−0.0128 (11)	0.0012 (11)	−0.0057 (11)
C17	0.0367 (14)	0.0243 (14)	0.0215 (13)	−0.0157 (11)	0.0054 (11)	−0.0039 (11)
C18	0.0387 (14)	0.0219 (14)	0.0272 (14)	−0.0118 (11)	−0.0013 (12)	−0.0011 (11)
C19	0.0350 (14)	0.0285 (14)	0.0255 (14)	−0.0141 (11)	−0.0034 (11)	−0.0032 (11)
C20	0.0357 (14)	0.0234 (13)	0.0281 (14)	−0.0141 (11)	−0.0020 (12)	−0.0082 (11)

### Geometric parameters (Å, °)

**Table:**

Co1—N1	2.178 (2)	C9—C10	1.380 (6)
Co1—N2^i^	2.175 (3)	C11—C12	1.375 (5)
Co1—O1	2.0416 (18)	C13—C14	1.509 (4)
Co1—O2^ii^	2.011 (2)	C14—C20	1.390 (4)
Co1—O3^iii^	2.1426 (19)	C14—C15	1.393 (4)
Co1—O4^iii^	2.228 (2)	C15—C16	1.389 (4)
O1—C13	1.265 (3)	C16—C17	1.502 (4)
O2—C13	1.249 (4)	C16—C18	1.381 (4)
O3—C17	1.261 (4)	C18—C19	1.406 (4)
O4—C17	1.252 (3)	C19—C20	1.387 (4)
N1—C1	1.340 (4)	C1—H1	0.9300
N1—C5	1.342 (4)	C2—H2	0.9300
N2—C10	1.326 (5)	C4—H4	0.9300
N2—C11	1.337 (4)	C5—H5	0.9300
N3—C19	1.388 (4)	C6—H6A	0.9700
N3—H3A	0.8600	C6—H6B	0.9700
N3—H3B	0.8600	C7—H7A	0.9700
C1—C2	1.375 (4)	C7—H7B	0.9700
C2—C3	1.386 (4)	C9—H9	0.9300
C3—C6	1.511 (5)	C10—H10	0.9300
C3—C4	1.388 (5)	C11—H11	0.9300
C4—C5	1.370 (4)	C12—H12	0.9300
C6—C7	1.489 (5)	C15—H15	0.9300
C7—C8	1.502 (5)	C18—H18	0.9300
C8—C12	1.373 (5)	C20—H20	0.9300
C8—C9	1.379 (5)		
			
Co1···C15^ii^	3.893 (3)	C19···C18^ix^	3.421 (4)
Co1···H15^ii^	3.1900	C1···H3A^iv^	2.7500
O1···O4^iii^	3.151 (3)	C2···H3A^iv^	2.8000
O1···N1	2.913 (3)	C4···H7A	2.7100
O1···N2^i^	3.111 (3)	C6···H6A^vi^	3.0900
O1···C5	2.995 (4)	C7···H4	2.8200
O1···C10^i^	3.246 (4)	C9···H6A	3.0000
O1···O2^ii^	3.235 (3)	C11···H15^vi^	3.0400
O1···C7^iv^	3.362 (5)	C12···H3B^xiii^	2.7500
O2···O1^ii^	3.235 (3)	C13···H10^i^	2.7500
O2···C10^i^	3.276 (5)	C17···H11^viii^	2.7400
O2···O3^v^	3.155 (3)	C18···H6B^xii^	2.9900
O2···N2^vi^	3.001 (4)	C19···H6B^xii^	2.9900
O2···C1^ii^	3.241 (4)	C20···H7B^iv^	3.0100
O2···N1^ii^	2.917 (3)	H1···O2^ii^	2.8800
O3···N1^vii^	2.988 (3)	H2···H6B	2.4000
O3···C1^vii^	3.269 (4)	H3A···H20	2.4200
O3···N2^viii^	3.060 (3)	H3A···C1^iv^	2.7500
O3···C11^viii^	3.082 (4)	H3A···C2^iv^	2.8000
O3···O2^v^	3.155 (3)	H3B···C12^xii^	2.7500
O4···C4^ix^	3.271 (4)	H3B···H6B^xii^	2.3300
O4···O1^vii^	3.151 (3)	H3B···H12^xii^	2.5400
O4···N2^viii^	3.116 (3)	H3B···H18	2.4400
O4···N1^vii^	3.099 (3)	H4···C7	2.8200
O1···H5	2.4600	H4···H7A	2.2300
O1···H20	2.5000	H4···O4^ix^	2.3500
O1···H10^i^	2.7100	H5···O1	2.4600
O1···H7A^iv^	2.8300	H5···H20	2.5700
O2···H15	2.5100	H6A···C9	3.0000
O2···H10^i^	2.4400	H6A···C6^vi^	3.0900
O2···H1^ii^	2.8800	H6A···H6A^vi^	2.3200
O3···H15	2.5900	H6B···N3^xiii^	2.6500
O3···H15^v^	2.5600	H6B···C18^xiii^	2.9900
O3···H11^viii^	2.4700	H6B···C19^xiii^	2.9900
O3···H11^vi^	2.8800	H6B···H2	2.4000
O4···H18	2.6500	H6B···H3B^xiii^	2.3300
O4···H4^ix^	2.3500	H7A···C4	2.7100
O4···H7A^ix^	2.6500	H7A···H4	2.2300
N1···O1	2.913 (3)	H7A···H9	2.5300
N1···O3^iii^	2.988 (3)	H7A···O1^iv^	2.8300
N1···O4^iii^	3.099 (3)	H7A···O4^ix^	2.6500
N1···C17^iii^	3.312 (4)	H7B···H12	2.4200
N1···O2^ii^	2.917 (3)	H7B···C20^iv^	3.0100
N2···O1^x^	3.111 (3)	H7B···H20^iv^	2.5000
N2···O3^xi^	3.060 (3)	H9···H7A	2.5300
N2···O4^xi^	3.116 (3)	H10···O1^x^	2.7100
N2···C17^xi^	3.282 (4)	H10···O2^x^	2.4400
N2···O2^vi^	3.001 (4)	H10···C13^x^	2.7500
N3···C16^ix^	3.405 (4)	H11···O3^xi^	2.4700
N3···H6B^xii^	2.6500	H11···C17^xi^	2.7400
C4···O4^ix^	3.271 (4)	H11···O3^vi^	2.8800
C7···O1^iv^	3.362 (5)	H12···H3B^xiii^	2.5400
C10···O2^x^	3.276 (5)	H12···H7B	2.4200
C10···C13^x^	3.543 (5)	H15···O2	2.5100
C11···C17^xi^	3.301 (4)	H15···O3	2.5900
C11···C15^vi^	3.524 (4)	H15···Co1^ii^	3.1900
C11···O3^xi^	3.082 (4)	H15···O3^v^	2.5600
C13···C10^i^	3.543 (5)	H15···C11^vi^	3.0400
C15···C11^vi^	3.524 (4)	H18···O4	2.6500
C15···Co1^ii^	3.893 (3)	H18···H3B	2.4400
C16···N3^ix^	3.405 (4)	H20···O1	2.5000
C17···C11^viii^	3.301 (4)	H20···H3A	2.4200
C17···N2^viii^	3.282 (4)	H20···H5	2.5700
C18···C18^ix^	3.573 (4)	H20···H7B^iv^	2.5000
C18···C19^ix^	3.421 (4)		
			
O1—Co1—N1	87.24 (8)	O1—C13—C14	117.0 (3)
O1—Co1—N2^i^	95.05 (9)	C13—C14—C15	119.3 (3)
O1—Co1—O3^iii^	154.55 (8)	C13—C14—C20	120.9 (3)
O1—Co1—O4^iii^	95.00 (8)	C15—C14—C20	119.7 (3)
O1—Co1—C17^iii^	124.90 (9)	C14—C15—C16	119.5 (3)
O1—Co1—O2^ii^	105.91 (8)	C17—C16—C18	120.5 (3)
N1—Co1—N2^i^	177.68 (9)	C15—C16—C18	120.6 (3)
O3^iii^—Co1—N1	87.51 (7)	C15—C16—C17	118.9 (3)
O4^iii^—Co1—N1	89.38 (8)	O3—C17—O4	121.1 (3)
N1—Co1—C17^iii^	89.68 (9)	O3—C17—C16	118.6 (3)
O2^ii^—Co1—N1	88.18 (8)	Co1^vii^—C17—O3	58.69 (14)
O3^iii^—Co1—N2^i^	90.27 (9)	O4—C17—C16	120.3 (3)
O4^iii^—Co1—N2^i^	90.08 (9)	Co1^vii^—C17—O4	62.59 (15)
N2^i^—Co1—C17^iii^	88.72 (10)	Co1^vii^—C17—C16	174.0 (2)
O2^ii^—Co1—N2^i^	91.52 (9)	C16—C18—C19	120.5 (3)
O3^iii^—Co1—O4^iii^	60.05 (7)	N3—C19—C18	120.9 (3)
O3^iii^—Co1—C17^iii^	30.19 (9)	N3—C19—C20	120.9 (3)
O2^ii^—Co1—O3^iii^	98.80 (8)	C18—C19—C20	118.3 (3)
O4^iii^—Co1—C17^iii^	29.93 (9)	C14—C20—C19	121.4 (3)
O2^ii^—Co1—O4^iii^	158.80 (7)	N1—C1—H1	118.00
O2^ii^—Co1—C17^iii^	128.97 (9)	C2—C1—H1	118.00
Co1—O1—C13	130.47 (19)	C1—C2—H2	120.00
Co1^ii^—O2—C13	148.9 (2)	C3—C2—H2	120.00
Co1^vii^—O3—C17	91.12 (17)	C3—C4—H4	120.00
Co1^vii^—O4—C17	87.48 (18)	C5—C4—H4	120.00
Co1—N1—C1	123.40 (18)	N1—C5—H5	118.00
Co1—N1—C5	120.13 (18)	C4—C5—H5	118.00
C1—N1—C5	116.3 (2)	C3—C6—H6A	109.00
C10—N2—C11	115.9 (3)	C3—C6—H6B	109.00
Co1^x^—N2—C10	121.2 (2)	C7—C6—H6A	109.00
Co1^x^—N2—C11	122.7 (2)	C7—C6—H6B	109.00
C19—N3—H3B	120.00	H6A—C6—H6B	108.00
H3A—N3—H3B	120.00	C6—C7—H7A	109.00
C19—N3—H3A	120.00	C6—C7—H7B	109.00
N1—C1—C2	123.5 (3)	C8—C7—H7A	109.00
C1—C2—C3	120.1 (3)	C8—C7—H7B	109.00
C2—C3—C4	116.3 (3)	H7A—C7—H7B	108.00
C2—C3—C6	122.1 (3)	C8—C9—H9	120.00
C4—C3—C6	121.5 (3)	C10—C9—H9	120.00
C3—C4—C5	120.3 (3)	N2—C10—H10	118.00
N1—C5—C4	123.5 (3)	C9—C10—H10	118.00
C3—C6—C7	114.5 (3)	N2—C11—H11	118.00
C6—C7—C8	113.7 (3)	C12—C11—H11	118.00
C9—C8—C12	116.0 (3)	C8—C12—H12	120.00
C7—C8—C12	122.5 (3)	C11—C12—H12	120.00
C7—C8—C9	121.6 (4)	C14—C15—H15	120.00
C8—C9—C10	120.1 (3)	C16—C15—H15	120.00
N2—C10—C9	124.0 (4)	C16—C18—H18	120.00
N2—C11—C12	123.2 (3)	C19—C18—H18	120.00
C8—C12—C11	120.8 (3)	C14—C20—H20	119.00
O2—C13—C14	118.6 (3)	C19—C20—H20	119.00
O1—C13—O2	124.4 (3)		
			
N1—Co1—O1—C13	−97.8 (3)	Co1^x^—N2—C10—C9	−177.1 (3)
N2^i^—Co1—O1—C13	82.6 (3)	C10—N2—C11—C12	0.9 (5)
O3^iii^—Co1—O1—C13	−176.1 (2)	Co1^x^—N2—C11—C12	177.1 (3)
O4^iii^—Co1—O1—C13	173.1 (3)	N1—C1—C2—C3	1.0 (5)
C17^iii^—Co1—O1—C13	174.5 (2)	C1—C2—C3—C4	0.2 (5)
O2^ii^—Co1—O1—C13	−10.5 (3)	C1—C2—C3—C6	179.9 (3)
O1—Co1—N1—C1	155.5 (2)	C2—C3—C4—C5	−0.9 (5)
O1—Co1—N1—C5	−29.6 (2)	C6—C3—C4—C5	179.4 (3)
O3^iii^—Co1—N1—C1	−49.4 (2)	C2—C3—C6—C7	133.5 (4)
O3^iii^—Co1—N1—C5	125.5 (2)	C4—C3—C6—C7	−46.8 (5)
O4^iii^—Co1—N1—C1	−109.5 (2)	C3—C4—C5—N1	0.6 (5)
O4^iii^—Co1—N1—C5	65.4 (2)	C3—C6—C7—C8	176.0 (3)
C17^iii^—Co1—N1—C1	−79.6 (2)	C6—C7—C8—C9	−80.7 (5)
C17^iii^—Co1—N1—C5	95.4 (2)	C6—C7—C8—C12	98.6 (4)
O2^ii^—Co1—N1—C1	49.5 (2)	C7—C8—C9—C10	178.5 (3)
O2^ii^—Co1—N1—C5	−135.6 (2)	C12—C8—C9—C10	−0.9 (5)
O1—Co1—N2^i^—C10^i^	−26.1 (3)	C7—C8—C12—C11	−178.4 (3)
O1—Co1—N2^i^—C11^i^	157.9 (2)	C9—C8—C12—C11	1.0 (5)
O1—Co1—O3^iii^—C17^iii^	−15.4 (3)	C8—C9—C10—N2	0.9 (6)
N1—Co1—O3^iii^—C17^iii^	−93.66 (18)	N2—C11—C12—C8	−1.0 (5)
O1—Co1—O4^iii^—C17^iii^	177.63 (18)	O1—C13—C14—C15	−170.9 (3)
N1—Co1—O4^iii^—C17^iii^	90.45 (18)	O1—C13—C14—C20	6.7 (4)
O1—Co1—C17^iii^—O3^iii^	172.00 (15)	O2—C13—C14—C15	10.6 (4)
O1—Co1—C17^iii^—O4^iii^	−2.9 (2)	O2—C13—C14—C20	−171.8 (3)
N1—Co1—C17^iii^—O3^iii^	85.58 (17)	C13—C14—C15—C16	178.8 (3)
N1—Co1—C17^iii^—O4^iii^	−89.30 (17)	C20—C14—C15—C16	1.2 (5)
O1—Co1—O2^ii^—C13^ii^	86.0 (4)	C13—C14—C20—C19	−179.3 (3)
N1—Co1—O2^ii^—C13^ii^	172.6 (4)	C15—C14—C20—C19	−1.7 (5)
Co1—O1—C13—O2	−25.5 (5)	C14—C15—C16—C17	−178.6 (3)
Co1—O1—C13—C14	156.1 (2)	C14—C15—C16—C18	1.0 (5)
Co1^ii^—O2—C13—O1	97.8 (4)	C15—C16—C17—O3	28.8 (4)
Co1^ii^—O2—C13—C14	−83.8 (4)	C15—C16—C17—O4	−150.4 (3)
Co1^vii^—O3—C17—O4	5.3 (3)	C18—C16—C17—O3	−150.7 (3)
Co1^vii^—O3—C17—C16	−173.9 (3)	C18—C16—C17—O4	30.1 (5)
Co1^vii^—O4—C17—O3	−5.1 (3)	C15—C16—C18—C19	−2.6 (5)
Co1^vii^—O4—C17—C16	174.1 (3)	C17—C16—C18—C19	176.9 (3)
Co1—N1—C1—C2	173.8 (2)	C16—C18—C19—N3	−179.2 (3)
C5—N1—C1—C2	−1.3 (4)	C16—C18—C19—C20	2.1 (5)
Co1—N1—C5—C4	−174.8 (2)	N3—C19—C20—C14	−178.7 (3)
C1—N1—C5—C4	0.5 (4)	C18—C19—C20—C14	0.1 (5)
C11—N2—C10—C9	−0.8 (5)		

Symmetry codes: (i) *x*−1, *y*, *z*+1; (ii) −*x*, −*y*+1, −*z*+1; (iii) *x*, *y*−1, *z*; (iv) −*x*, −*y*+1, −*z*; (v) −*x*, −*y*+2, −*z*+1; (vi) −*x*+1, −*y*+1, −*z*; (vii) *x*, *y*+1, *z*; (viii) *x*−1, *y*+1, *z*+1; (ix) −*x*, −*y*+2, −*z*; (x) *x*+1, *y*, *z*−1; (xi) *x*+1, *y*−1, *z*−1; (xii) *x*−1, *y*+1, *z*; (xiii) *x*+1, *y*−1, *z*.

### Hydrogen-bond geometry (Å, °)

**Table:**

Cg4 is the centroid of the N1-pyridine ring.

**Table:**

*D*—H···*A*	*D*—H	H···*A*	*D*···*A*	*D*—H···*A*
C4—H4···O4^ix^	0.93	2.35	3.271 (4)	173
C5—H5···O1	0.93	2.46	2.995 (4)	117
C10—H10···O2^x^	0.93	2.44	3.276 (5)	150
C11—H11···O3^xi^	0.93	2.47	3.082 (4)	124
C15—H15···O3^v^	0.93	2.56	3.487 (4)	175
N3—H3A···Cg4^iv^	0.86	2.92	3.765 (3)	169
C11—H11···Cg3^xi^	0.93	2.65	3.019 (3)	105

Symmetry codes: (ix) −*x*, −*y*+2, −*z*; (x) *x*+1, *y*, *z*−1; (xi) *x*+1, *y*−1, *z*−1; (v) −*x*, −*y*+2, −*z*+1; (iv) −*x*, −*y*+1, −*z*.
